# Monopolizing Sanctioning Power under Noise Eliminates Perverse Punishment But Does Not Increase Cooperation

**DOI:** 10.3389/fnbeh.2016.00180

**Published:** 2016-09-29

**Authors:** Sven Fischer, Kristoffel Grechenig, Nicolas Meier

**Affiliations:** ^1^Newcastle University, Newcastle University Business SchoolNewcastle upon Tyne, UK; ^2^Max Planck Institute for Research on Collective GoodsBonn, Germany; ^3^School of Business and Economics, RWTH Aachen UniversityAachen, Germany

**Keywords:** cooperation, public good, centralized punishment, imperfect information, anti-social punishment, perverse punishment

## Abstract

We run several experiments which allow us to compare *cooperation* under *perfect* and *imperfect information* in a *centralized* and *decentralized punishment* regime. Under perfect and extremely noisy information, aggregate behavior does not differ between institutions. Under intermediate noise, punishment escalates in the decentralized peer-to-peer punishment regime which badly affects efficiency while sustaining cooperation for longer. Only decentralized punishment is often directed at cooperators (perverse punishment). We report several, sometimes subtle, differences in punishment behavior, and how contributions react.

## 1. Introduction

Modern societies have centralized sanctioning power as a means to enforce norms (Weber, 1919). This monopoly has often been justified on the premise that private, decentralized enforcement has (higher) negative externalities (Clotfelter, 1978; Polinsky, 1980). But, is centralization necessarily better? Experiments on voluntary cooperation repeatedly demonstrate that, compared to an environment *without punishment*, decentralized, informal, peer-to-peer punishment increases cooperation under perfect information (Yamagishi, 1986; Ostrom et al., 1992; Fehr and Gächter, 2000, 2002), and welfare in the long run (Gächter et al., 2008). Various studies, however, challenge the idea that peer-to-peer punishment generally enhances cooperation (for an overview, see Nikiforakis, 2014): for example on the basis of punishment which is targeted at cooperators, referred to as anti-social (Herrmann et al., 2008) or perverse punishment (Cinyabuguma et al., 2006), or on the basis of counter-punishment (targeted either at the group or the punisher directly as in Nikiforakis, 2008; Nikiforakis et al., 2012)^1^. If punishment is centralized, however, there is no opportunity for counter punishment and such sources of inefficiency are less likely. Furthermore, with only one punisher, there are no coordination problems and no problems resulting from possibly conflicting contribution norms. Some experiments test the effectiveness of *formal, centralized* enforcement mechanisms compared to informal, decentralized regimes, while capturing important aspects of institutions. This literature characterizes centralization as a mechanism that allows to commit to a sanctioning scheme, such that punishment is automatically carried out and/or determined according to some exogenous voting rule (Kosfeld and Riedl, 2004; Tyran and Feld, 2006; Guillen et al., 2007; Kube and Traxler, 2011; Putterman et al., 2011; Andreoni and Gee, 2012; Ambrus and Greiner, 2015). Centralization is viewed as a commitment mechanism and these papers do not explore the effect of centralization *per se*, i.e., of merely concentrating all power into one hand.

In an environment with a centralized punisher rather than an automated mechanism, an important question is how the authorization to punish is granted. Baldassarri and Grossman (2011) compare two centralized punishment regimes. In one, authority is granted by chance, in the other by electing the punisher from within the group after two trial rounds. While punishment behavior is very similar, in the elected authority treatment participants cooperate more. This suggests that in order to test whether centralization *per se* matters, one needs to abstract from any mechanism that grants legitimacy, and restrict the analysis to a fair random selection. There are a few studies which allow to compare a centralized with a decentralized punishment regime in this way, and the resulting evidence is mixed. In Carpenter et al. (2012), where the role of the punisher is randomly allocated but fixed and punishment is cheap, contributions and overall efficiency are larger in the decentralized regime. Similarly, in Nosenzo and Sefton (2014) contributions are substantially larger in a mutual punishment regime than in a centralized one. In O'Gorman et al. (2009), on the other hand, where authority is also granted at random but changes every round, and where punishment is expensive, there are no significant differences in cooperation. However, due to significantly more punishment, the decentralized regime is less efficient.

One conclusion from these studies is that the effectiveness of a centralized regime stands and falls with the ability and willingness of just one person to invest resources for punishment, which results in considerably more variability in performance between groups. Decentralized regimes, on the other hand, suffer from the problems already mentioned. Such negative effects are likely to be aggravated by noisy information, which so far was only tested in decentralized regimes. Several studies show that peer to peer punishment is not able to sustain cooperation under imperfect information (Grechenig et al., 2010; Ambrus and Greiner, 2012; Grechenig et al., 2015) and it remains unclear whether this equally holds for centralized punishment. For example, imperfect information may result in some to stop punishing altogether, even irrespective of total group contributions. While in a decentralized regime this could partly be compensated by more punishment of others, if punishment is centralized, this is not possible.

In order to test the effect of centralization *per se* under different degrees of *imperfect information*, we hold all other considerations constant across institutions: (1y, (1) punishers cannot commit to punishment ex ante, (2) contributors cannot influence who is allowed to punish, (3) the direct consequences from punishment are the same in both institutions, and (4) there are no differences in externalities from punishment. We introduce noise in the signals about individual contributions in order to test whether decentralized or centralized punishment is more robust to imperfect information. By abstracting from institutional factors, we return to the origins of formal punishment as a centralization of informal sanctioning regimes (Turnbull, 1962; Guala, 2012). Furthermore, by allowing participants to interact over 30 periods, we obtain enough observations for a detailed analysis of individual behavior and group dynamics.

To the best of our knowledge, we are the first to analyze the effects of centralization of punishment *per se* under *imperfect information*.

## 2. Experimental design

We use a standard finitely repeated linear public-good game with a voluntary contribution mechanism. Participants interact in groups of five over 30 periods in a partner design, where every period has two stages, a contribution and a punishment stage. In our set of experiments we have two treatments. In the first we compare two different punishment *institutions*: Decentralized (*DEC*) and Centralized punishment (*CEN*). In the second we control for the accuracy of the signal about the contributions in the group. More specifically, we compare a perfect signal with three different levels of noise, indicated by the probability λ of an accurate signal with λ = 1, λ = 0.75, or λ = 0.50. Thus, the signal is either always accurate, or only 75 or 50% of the time. In the following we identify a treatment condition by the combination of institution and λ. For example DEC/1 is the treatment with decentralized punishment and a perfect signal.

There are five participants in every group, four *C-participants* (*i* ∈ {1…4}) and one *Authority* (*A*), and every period consists of two stages, a contribution and a punishment stage. In the first stage, the contribution stage, the four *C-participants* can contribute to the public good; the remaining participant, the *Authority* (*A*), benefits from the public good but cannot contribute herself. After the contribution stage, all five participants first receive a common signal about the contributions, where the quality of the signal depends on λ. Then, in treatments with centralized punishment (CEN) the authority decides over punishment. In treatments with decentralized punishment (DEC) this is done by the C-participants and the Authority is merely passive. More specifically, she cannot influence punishment but is, nevertheless, affected by the contribution and punishment decisions of the four C-participants. In the following we describe each stage in more detail^2^.

### 2.1. Contribution stage

In the first stage of each of the 30 rounds, each of the four C-participants receives an endowment of *e*_*g*_ = 20 tokens. They then simultaneously and independently determine their contribution to the public good *g*_*i*_ with *g*_*i*_ ∈ *G* = {0, 2, 4, …, 20}

In line with most experiments on decentralized punishment in public good games, we chose a marginal per capita return of 0.4. Hence, the monetary payoff of player *i* in the first stage is given by

(1) $$\begin{array}{l} \pi_{i}^{1}=e_{g}-g_{i}+0.4\underset{k}{\sum }g_{k} \end{array}$$

The authority *A*, despite not contributing, equally benefits from the public good:

(2) $$\begin{array}{l} \pi_{A}^{1}=0.4\underset{k}{\sum }g_{k} \end{array}$$

### 2.2. Punishment stage

In the second stage, every C-participant *i* and authority *A* receives the same signal *s*_*k*_ about the contributions *g*_*k*_ of participant *k* with *k* ≠ *i*. The signal itself is independently determined for every contribution in the group. With probability λ the signal is correct and *s*_*k*_ = *g*_*k*_, with probability 1−*p* the signal is incorrect. An incorrect signal is taken from the uniform distribution of all possible contributions excluding the actual one. More specifically,

$$s_{k}=\{\begin{array}{l} g_{k}\text{ with probability}=\lambda \\ {\tilde{g}}_{k}\text{ with probability}=1-\lambda \end{array},$$

where λ is the accuracy of the signal and ${\tilde{g}}_{k}$ is an independent realization out of the uniform distribution *G* \ {*g*_*k*_}. As all participants receive the same signals about contributions of others, information conditions for punishers are constant across treatments. Note, however, that every C-participant always knows for sure how much he contributed himself but they do not know which signal others receive about their own contribution^3^.

C-participants were identified by numbers 1–4 which were assigned randomly anew every period and made identification of group members difficult. We contrast a perfect signal (λ = 1) with three levels of noise: λ = 0.5 and λ = 0.75. For example, for λ = 0.5, a contribution of 6 would lead to the signal “6” with probability 0.5, and to any other signal, each with probability 0.05 (0.5/10). Thus, despite considerable noise, a signal is still informative.

#### 2.2.1. Punishment

In both punishment institutions, each of the four C-participants *i* receives a punishment endowment of *e*_*p*_ = 10 punishment points, and authority *A* receives an additional endowment of $e_{p}^{A}=40$. Otherwise the rules differ.

#### 2.2.2. Punishment in *DEC*

In *DEC* the four C-participants can distribute punishment points amongst each other, where each point costs them one unit and also reduces the authority's income by one^4^. Every received punishment point reduces the target participant's income by three units. More specifically, denoting a punishment point sent by *i* to *j* with *p*_*ij*_, the total payoff of participant *i* is:

(3) $$\pi_{i}=\pi_{i}^{1}+e_{p}-\sum_{j}p_{ij}-3\sum_{j}p_{ji}\text{ ,}$$

and the payoff of participant *A* is:

(4) $$\begin{array}{l} \pi_{A}=\pi_{A}^{1}+e_{p}^{A}-\underset{i}{\sum }\underset{j\neq i}{\sum }p_{ij} \end{array}$$

We include the authority *A* in treatment *DEC* as a passive participant in order to hold considerations, such as the externalities from punishment (see Engel and Rockenbach, 2009), constant across treatments.

#### 2.2.3. Punishment in *CEN*

In *CEN*, only authority *A* can distribute punishment points. Every punishment point distributed by *A* reduces *A*'s payoff by one, the punished subject's payoff by three, and the payoff of each other participant by 1/3. This keeps the overall costs of punishment constant across treatments (participants finance the punishment applied by *A*, except one's own punishment). Furthermore, we kept the maximal amount of punishment points that could be assigned to one participant constant across treatments by limiting *A* to distributing at most 30 points to one individual. Thus, in *CEN*, final payoffs are determined as follows:

(5) $$\pi_{i}=\pi_{i}^{1}+e_{p}-\frac{1}{3}\sum_{j\;\neq \;i}p_{Aj}-3p_{Ai}\text{ ,}$$

and

(6) $$\begin{array}{l} \pi_{A}=\pi_{A}^{1}+e_{p}^{A}-\underset{j}{\sum }p_{Aj}\;, \end{array}$$

where equivalently to *p*_*ij*_ we denote with *p*_*Aj*_ the number of punishment points assigned to *j* by *A*. This payoff structure keeps everything constant across institutions, including externalities, efficiency concerns, commitment, and punishment power, such that we exclusively explore the effect of centralization *per se*.

#### 2.2.4. Information at the end of one round

At the end of a round, every participant is informed about his income from the public good, the amount of punishment received, and his final total payoff. Thus, any inaccurate perception about one's own income, resulting from noisy signals about group contributions, are resolved at the end of every round.

### 2.3. Equilibrium

To find the set of equilibria under the assumptions of selfishness, common knowledge of rationality and sequential rationality, we first look at the stage game. As punishment is costly, it is strictly dominated, both for *A* in *CEN*, as well as for the regular participants in *DEC*. Thus, play at the contribution stage remains unaffected and zero contribution by all remains the unique equilibrium in dominant strategies. As zero contribution and no punishment guarantees the minmax payoff, the equilibrium is not affected by the finite number of repetitions, and there should be no cooperation and no punishment throughout the 30 periods. This rationale applies to all treatments, such that there should be no differences between different punishment institutions and different levels of noise.

### 2.4. Setup

We use a 2 × 3 factorial design between subjects, i.e., every subject participates in only one of our six treatment combinations. Subjects interact repeatedly over 30 periods in a partners design, i.e., groups are kept constant. All rounds are paid.

The experiments were run in the experimental laboratory of the University of Bonn (EconLab) and was programmed and conducted with the software z-Tree (Fischbacher, 2007). We ran a total of 12 sessions with 240 (20 per session) student participants (mostly undergraduate from various fields of study) divided into 48 groups (8 groups of 5 participants per treatment), and no participant took part in more than one session. We relied on ORSEE (Greiner, 2015) for recruitment.

For 100 experimental *Taler* participants earned €1. Sessions lasted for about 90 min (including admission and payment) and participants earned on average €14.28 (including a show up fee of €2.50), about USD 18.80, which is more than the usual hourly wage for student jobs.

## 3. Results

We first report aggregate results with respect to institutions and noise before we analyze punishment behavior, the resulting incentives, and intertemporal reactions in more detail.

### 3.1. Aggregate outcomes

**Figure 1:**
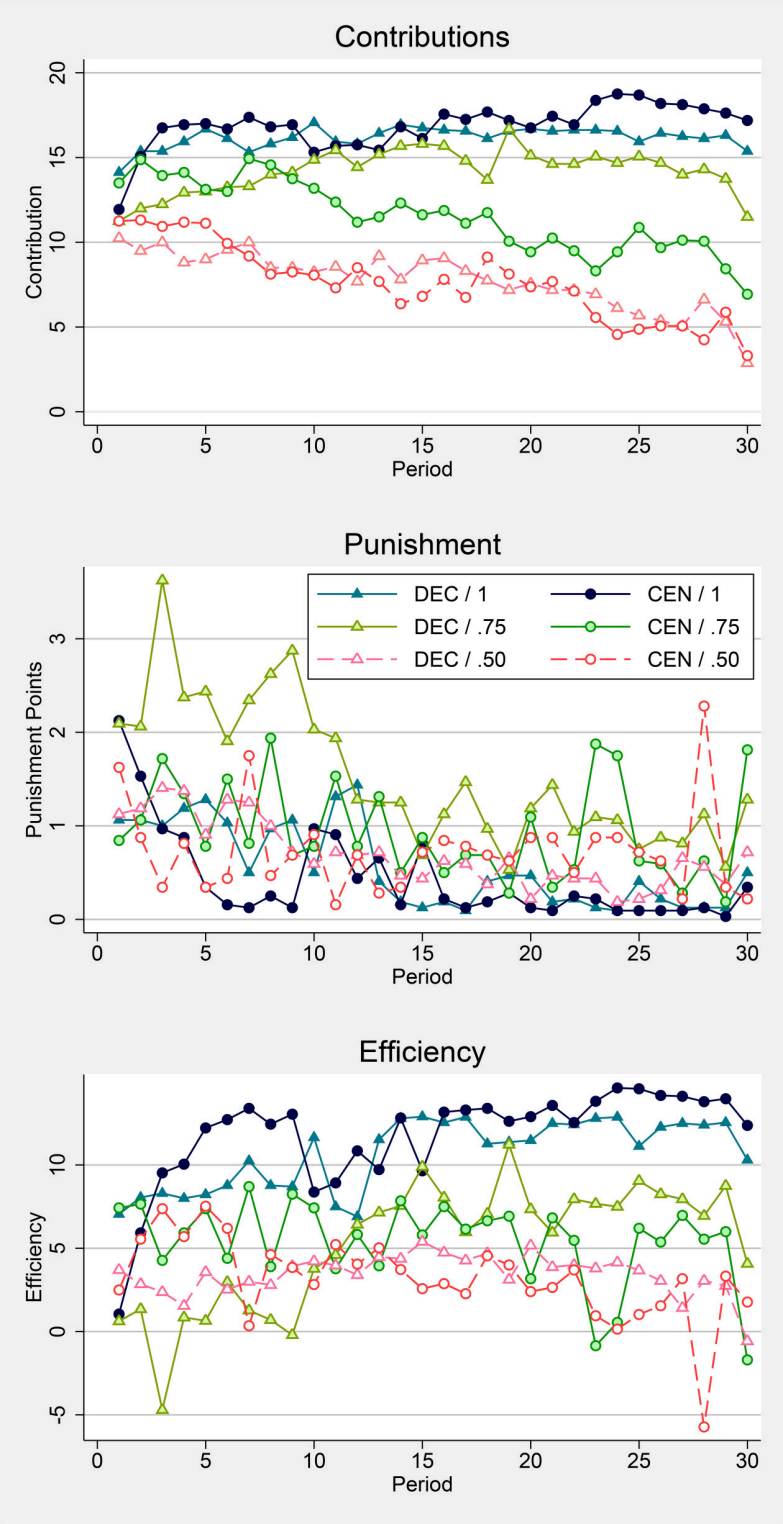
**Outcomes**. Treatments with DECentralized or CENtralized punishment and a probability of a correct signal about individual contributions of 1, 0.75, or 0.50.

We compare contributions, overall punishment, and efficiency between institutions and noise levels. The graphs in Figure 1 show average contributions, punishment points (distributed to C-participants), and efficiency over time across treatment combinations. Efficiency is measured as the difference between total earnings minus fixed endowments (for contribution and punishment stage), including authorities. Thus, an efficiency of 0 means that inefficiencies due to punishment balance out gains from cooperation.

#### 3.1.1. Differences between institutions

Table 1 reports the *p*-values of two-sided Wilcoxon rank-sum tests comparing the distributions of matching group averages between DEC and CEN within each noise condition (see rows)^5^. For each variable of interest, contributions, punishment points, and total efficiency, we test separately for the first (Periods 1–15) and second half (Periods 16–30) of the experiment (see columns) and rely on acceptance thresholds of 10%.

**Table 1:** **Aggregate effects of institution**.

	**Contrib**.		**Punishment**		**Efficiency**	
**Periods:**	**1–15**	**16–30**	**1–15**	**16–30**	**1–15**	**16-30**
λ = 1	0.752	0.709	0.713	0.594	0.834	0.709
λ = 0.75	0.753	**0.046**	**0.027**	0.462	0.142	0.142
λ = 0.50	0.495	0.674	0.793	0.400	0.753	0.529

*Each cell reports the p−value of a Wilcoxon rank-sum test comparing the distributions of averages per matching group between CEN and DEC for a given λ (row). Averages per matching group are either taken over first or last 15 periods. Significant outcomes with p < 0.1 are highlighted*.

For a perfect signal, i.e., whenever λ = 1 there are neither significant differences in contributions nor in punishment or efficiency. Similarly, for considerable noise of λ = 0.50 Table 1 does not report any significant differences between institutions. For λ = 0.75, however, we find significant differences between institutions. In the second half of the experiment contributions are significantly higher in DEC/0.75 than in CEN/0.75. Furthermore, there is significantly more punishment in DEC than in CEN in the first half of the experiment, but not in the second half. The substantially higher punishment in DEC/0.75 in the first half, and here especially in the first ten periods, results in significantly lower efficiency than in CEN/0.75. Despite contributions being higher in late rounds, there are no significant differences in efficiency. If instead of total efficiency we only look at *Profits* of C-participants, results are very similar and qualitatively identical for all levels of λ.

Result 1. *If signals are perfect* (λ = 1*) or highly distorted (*λ = 0.5*) aggregate outcomes do not differ significantly between institutions. For* λ = 0.75 *there is significantly more punishment and less efficiency in DEC early in the experiment, and in later periods contributions remain significantly higher in DEC/0.75 than in CEN/0.75*.

Most contributions in CEN/1 and DEC/1 are close to the ceiling of 20. In Section A.2. (Supplementary Material) of the online supplement we report simulations which show that despite of this we could have observed significant effects, if in DEC/1 a sufficient number of participants had contributed fully by default. For example, suppose that participants in DEC/1 had contributed fully with a baseline chance of 70 or 80%. In this case we could have found a significant difference between DEC/1 and CEN/1 at the 5% significance level with a probability of 52.3 and 94.6%, respectively.

#### 3.1.2. Effect of noise

Comparisons between different levels of noise within each institution mirror our previous results. Table 2 lists the *p*-values of Wilcoxon rank sum tests comparing outcomes between different levels of noise within each institution. Again we distinguish between the first and second half of the experiment (see columns).

**Table 2:** **Aggregate effects of noise**.

	**DEC**		**CEN**	
**Periods:**	**1–15**	**16–30**	**1–15**	**16–30**
	**Contributions**			
λ = 1 vs. 0.75	0.208	**0.059**	**0.092**	**0.010**
λ = 1 vs. 0.50	**0.009**	**0.012**	**0.021**	**0.002**
λ = 0.75 vs. 0.5	**0.059**	**0.002**	**0.059**	**0.074**
	**Punishment**			
λ = 1 vs. 0.75	**0.046**	**0.016**	0.401	**0.026**
λ = 1 vs. 0.50	1.00	0.342	0.916	**0.023**
λ = 0.75 vs. 0.5	**0.021**	0.103	0.431	0.916
	**Efficiency**			
λ = 1 vs. 0.75	**0.059**	**0.074**	**0.074**	**0.003**
λ = 1 vs. 0.50	**0.059**	**0.021**	**0.016**	**0.002**
λ = 0.75 vs. 0.5	0.600	**0.005**	0.401	0.294

*Each cell reports the p−value of a Wilcoxon rank-sum test comparing distributions of averages per matching group between different levels of noise within an institution. Averages per matching group are either taken over first or last 15 periods. Significant outcomes with p < 0.1 are in bold*.

In CEN *contributions* are always significantly smaller the more noisy the signal. In DEC, however, there are no significant differences between λ = 1 and λ = 0.75 in the first half of the experiment, while otherwise comparative statics are as in CEN. Aggregate *Punishment*, on the other hand remains unaffected by noise in CEN in the first half, but not in the second, where it is smaller in CEN/1 than in CEN/0.75 and CEN/0.50. In DEC only λ = 0.75 stands out. While punishment never differs between DEC/1 and DEC/0.50, there is significantly more punishment in DEC/0.75 than in in either DEC/1 or DEC/0.50 in the first half and significantly more than in DEC/1 also in the second half. In both institutions, *efficiency* is similarly affected by noise in the first half of the experiment. It is significantly higher for λ = 1 than for λ = 0.75 or λ = 0.50. While there are no significant differences between CEN/0.75 and CEN/0.50 throughout the experiment, efficiency is significantly higher for DEC/0.75 than for DEC/0.50 in the second half, a consequence of the higher contributions towards the end of DEC/0.75. In summary we find an interaction effect between institution and noise:

Result 2. *Irrespective of institution, noise has a negative effect on contributions in the long run. The effect of noise on punishment and efficiency, however, is non-monotonic and differs between institutions. Under centralized punishment CEN/1 stands out with significantly less punishment and higher efficiency in the long run, while there are hardly any differences between CEN/0.75 and CEN/0.50. Under decentralized punishment, DEC/0.75 stands out with more punishment and less efficiency especially in early rounds*.

### 3.2. Distributed punishment

We turn to regression analysis to identify how the decision to punish is correlated to signals about contributions. The columns in Table 3 report the results of estimations of the distributed amount of punishment points on characteristics of the received signals, separately for every treatment^6^. Variable *D*Free is a dummy variable indicating a signal of no contribution. Variables *D*aboveGroup and *D*belowGroup are dummy variables indicating that, according to the signal, the receiver of punishment has contributed more than the group average and less, respectively. In CEN we calculate the average group contribution as the average signal. In DEC we take the average over one's own contribution and the signals about the contributions of the other three. Similarly, variables *D*more and *D*less in treatment DEC are dummy variables indicating that, according to the signal, the (potential) receiver of punishment contributed more than the one who decides whether to punish and less, respectively. The definitions of *D*more, *D*less, *D*aboveGroup, and *D*belowGroup all exclude the case of no difference (avoiding perfect collinearity)^7^. Our measurement of cooperativeness as the deviation to the group average is similar to, for example, (Cinyabuguma et al., 2006). The measures based on individual comparisons in DEC are as in Herrmann et al. (2008). Clearly, both measures are related^8^.

**Table 3:** **Distributed punishment**.

	λ = 1			λ = 0.75			λ = 0.50		
	**DEC**		**CEN**	**DEC**		**CEN**	**DEC**		**CEN**
**DISTRIBUTED PUNISHMENT POINTS**									
Signal	−0.0333	≫	−0.127^***^	−0.0731^***^	≫	−0.105^***^	−0.134^***^	≪	−0.0511
	(−0.48)		(−11.73)	(−3.36)		(−6.13)	(−4.83)		(−0.72)
*D*Freerider	0.792^**^	≫	−0.110	0.0223	<	0.256	0.134	≺	0.494
	(2.54)		(−0.29)	(0.09)		(0.73)	(0.78)		(1.41)
*D*belowGroup	2.141^***^	≪	3.104^***^	1.191^***^		1.029	0.699^*^	<	1.400
	(3.12)		(5.13)	(5.68)		(1.30)	(1.96)		(1.38)
*d*aboveGroup	1.653^***^	>	0.503	0.168		0.117	0.496^**^	<	1.468
	(2.89)		(0.62)	(0.71)		(0.17)	(2.06)		(1.52)
*D*less	2.056^***^			1.792^***^			0.606^***^		
	(5.39)			(8.63)			(2.92)		
*D*more	1.689^***^			1.297^***^			0.737^***^		
	(4.01)			(6.96)			(5.92)		
Period	0.138	<	0.478^***^	0.281	<	0.437^*^	−0.206	≫	−0.772
	(0.43)		(3.21)	(1.28)		(1.85)	(−0.88)		(−1.43)
_cons	−5.080^***^	≪	−1.843^**^	−3.316^***^	≪	−1.116	−2.643^***^		−0.260
	(−8.93)		(−2.16)	(−5.15)		(−0.96)	(−3.55)		(−0.15)
N (#Groups)	2880 (8)		960 (8)	2880 (8)		960 (8)	2880 (8)		960 (8)
ll	−935.8		−393.7	−1940.2		−1137.1	−1141.8		−1078.8
Wald chi2	645.0^***^		954.5^***^	1333.3^***^		946.0^***^	513.5^***^		385.0^***^

Poisson regression of distributed punishment points with random shift effect on matching group and subject (DEC only, random effects independent). Not reported: fixed effect on every Period (following Period 2). t statistics in parentheses based on Huber White standard errors (not for Wald chi2).

* p < 0.1,

** p < 0.05,

*** *p < 0.01. Significance of differences between institutions (based on regression of data on both institutions, including dummy for treatment CEN and interaction effects): ≺ (p < 0.1), < (p < 0.05), ≪ (p < 0.01)*.

All regressions are maximum likelihood Poisson estimations which besides the above mentioned fixed effects have a random intercept effect per matching group, and in DEC also per participant (independent of group effect). Estimations also include fixed effects for periods (following period 2). We use Poisson estimations as this best controls for the skewed distribution of integer punishment points.

While in DEC patterns of punishment decisions are similar for all levels of λ, in CEN punishment becomes fairly undirected with increasing noise. In all treatment combinations, punishment decreases with increasing Signal about the contribution. Only in DEC/1 and CEN/0.50 this is not significant. Of all treatment combinations, only DEC/1 has significant additional punishment of free rider behavior (*D*Freerider). In all treatments someone gets punished more when the signal about his contribution is below the group average (*D*belowGroup), an effect which is significant in all treatments except CEN/0.75 and CEN/0.50. Similarly, participants who contribute less than the punisher (*D*less) in DEC are punished significantly more.

In DEC/1 and DEC/0.50 we furthermore find significant perverse punishment as measured by *D*aboveGroup, an effect which is absent in all CEN treatments. Furthermore, in all DEC treatments there is significant anti-social punishment as indicated by *D*more.

Result 3. *Distributed punishment*

- *becomes less systematic with increasing noise in CEN*,
- *and decreases with increasing signal in all treatments*.
- *There is perverse punishment in DEC/1 and DEC/0.5 but in none of the CEN treatments*.
- *There is anti-social punishment in DEC for all levels of* λ.

We furthermore tested whether there are different patterns in punishment behavior between the first 10, 15, last 15, and last 20 periods, especially in DEC/0.75. While there are some minor differences in the size of reactions, the overall patterns are similar. We furthermore tested whether differences in coefficients between, for example DEC/1 and CEN/1 are significant^9^. Significant differences are indicated by symbols such as ≻ (*p* < 0.1), > (*p* < 0.05), or ≫ (*p* < 0.01) between the two columns.

Result 4. *With only a few exceptions, punishment behavior differs significantly between institutions*.

Finally, which effect is stronger, punishment of negative or positive deviators?^10^. With the exception of DEC/0.50, punishment of negative deviators is stronger. Wald tests comparing coefficients *D*below with *D*above or *D*less with *D*more confirm that these differences are significant in treatments with λ = 1 or λ = 0.75 (all *p*-values below 0.0451), but not for λ = 0.50 (*p* ≥ 0.2073)

Result 5. *Except for treatment combinations with* λ = 0.50*, punishment of negative deviators is significantly stronger than that of positive deviators*.

### 3.3. Incentives

To adequately compare institutions we must compare how punishment is being experienced. In treatment DEC recipients of punishment can not identify individual punishers and therefore whether the punisher contributed more or less. Thus, contrary to distributed punishment, *D*more and *D*less are now undetermined in both institutions, which puts the two back on equal footing. Furthermore, for DEC it is conceivable that due to the many repetitions, behavior of “sophisticated” punishers may add a non-random component to how distributed punishment is aggregated in the group^11^. So, while in CEN received punishment must be equivalent to the way punishment is distributed, in DEC received punishment may be more than just a combination of distributed punishment with white noise.

Table 4 reports results of Poisson regressions of the received punishment on various characteristics of a participant's contribution. All regressors are now based on actual contributions rather than signals. This includes the definition of the group average for the calculation of *D*aboveGroup and *D*belowGroup.

**Table 4:** **Received punishment**.

	λ = 1			λ = 0.75			λ = 0.50		
	**DEC**		**CEN**	**DEC**		**CEN**	**DEC**		**CEN**
**DEDUCTED INCOME**									
Contribution	−0.0385	≫	−0.140^***^	−0.0360^**^	≪	0.00296	−0.0616^***^	≪	−0.0193
	(−0.61)		(−5.71)	(−2.28)		(0.17)	(−5.21)		(−0.63)
*D*Freerider	0.904^***^	≫	0.139	−0.117	≪	1.083^***^	0.281^*^		0.446
	(2.80)		(0.33)	(−0.46)		(5.73)	(1.70)		(1.32)
*D*belowGroup	3.511^***^	≫	2.871^***^	0.696^**^		0.597^**^	−0.196	<	0.408^**^
	(5.98)		(4.58)	(2.28)		(2.23)	(-0.22)		(2.45)
*D*aboveGroup	2.852^***^	≫	0.604	−0.103		−0.111	0.0945		0.268
	(7.02)		(0.70)	(−0.42)		(−0.49)	(0.12)		(0.98)
Period	0.035	≫	0.00272	0.0106		−0.0171	−0.067	≫	−0.116^***^
	(0.62)		(0.19)	(0.53)		(−0.40)	(−1.64)		(−3.03)
_cons	−2.172^**^	≪	−0.023	1.734^***^	≫	0.045	1.01		0.727
	(−2.29)		(−0.02)	(4.96)		(0.09)	(0.87)		(1.22)
N (#Groups)	960 (8)		960 (8)	960 (8)		960 (8)	960 (8)		960 (8)
Wald chi2	1325.2^***^		2154.5^***^	1594.7^***^		1634.1^***^	607.7^***^		787.8^***^
ll	−1381.3		−806.9	−4785.8		−3521.4	−2090.1		−2789.4

Poisson regression of deducted income with random shift effect on subject (DEC only) and matching group (independent). Not reported: fixed effect on every Period (following Period 2). t statistics in parentheses based on Huber White standard errors (not for Wald chi2).

* p < 0.1,

** p < 0.05,

*** *p < 0.01. Significance of differences between institutions (based on regression of data on both institutions, including dummy for treatment CEN and interaction effects): ≺(p < 0.1), < (p < 0.05), ≪ (p < 0.01)*.

For perfect signals our estimations by and large confirm our previous results^12^. Again we find significant punishment of negative deviators in both CEN/1 and DEC/1, and of positive deviators (*D*aboveGroup) in DEC/1 only. Contrary to our previous results, neither in DEC/0.75 nor in DEC/0.50 do participants experience significant perverse punishment. Despite the noise, in all treatments except for DEC/0.50, negative deviators receive significantly more punishment. Finally, there is no experience of perverse punishment in any of the CEN treatments.

Result 6. • *In DEC/1 negative and positive deviators experience punishment*.

- *In DEC/0.75 only negative deviators experience punishment*.
- *In DEC/0.50 received punishment is independent of how one's contribution relates to those in the group*.
- *In all CEN treatments negative deviators experience significantly more punishment while positive deviators do not*.

Again we compared coefficients between institutions. Significant results are indicated by symbols such as > between columns in Table 4. Similarly to our results on distributed punishment we find:

Result 7. *With only a few exceptions, experienced punishment differs significantly between institutions*.

**Figure 2:**
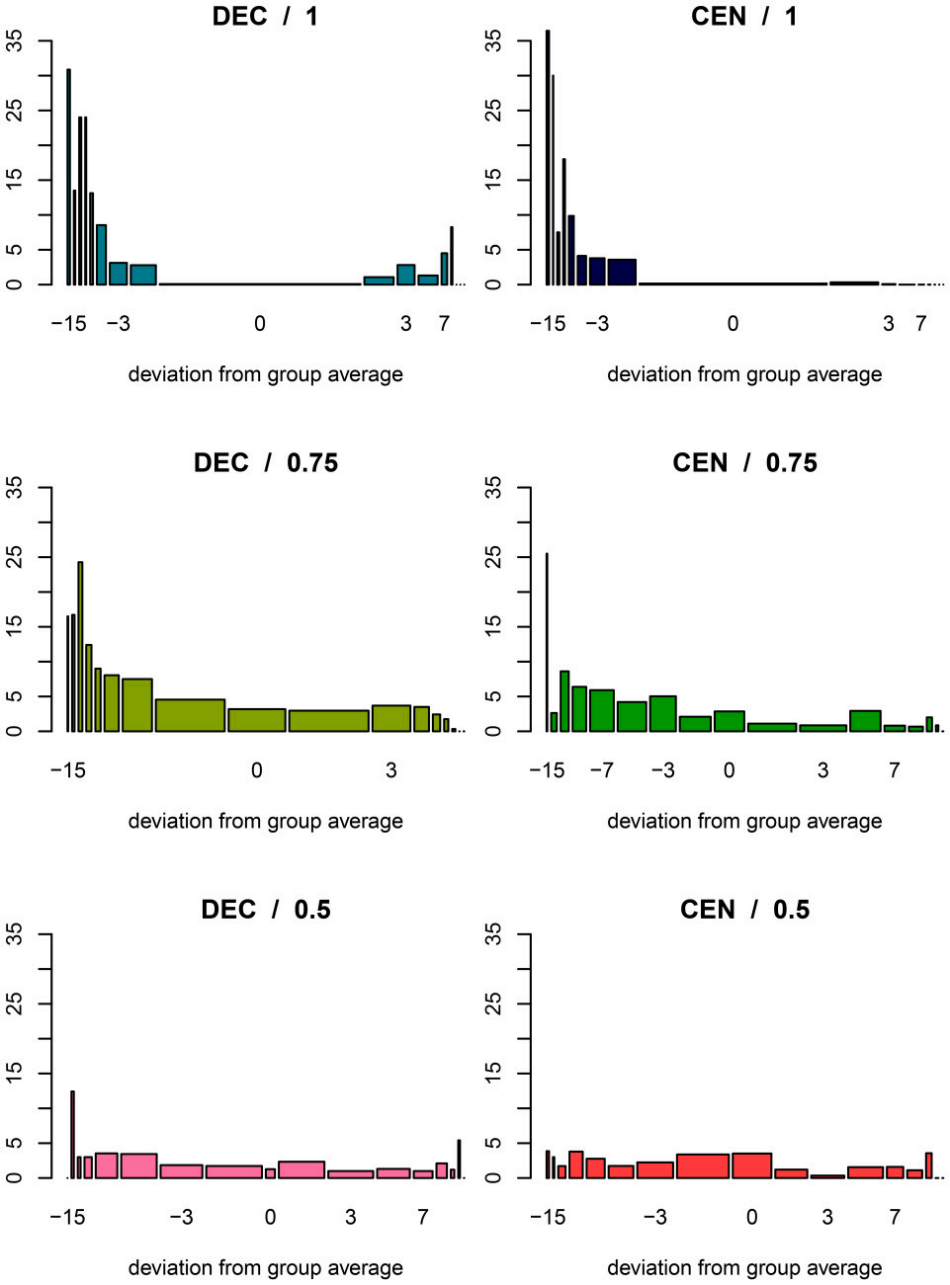
**Received punishment**. Barplot of average deducted income by difference between own contribution and average group contribution. Except for 0, categories show intervals of length 2: “−16 to −14”, “−14 to 12” …“−2 to < 0”, “0”, “>0–2”, …“14–16”. Width of bars is proportional to number of observations.

In addition to the regressions reported in Table 4 we ran estimations including variables *above* and *below* which measure the absolute distance of the contribution to the group average (rather than merely indicator variables *D*aboveGroup and *D*belowGroup). We report detailed results in the online supplement. The barplots in Figure 2 illustrate the average amount of punishment participants received conditioned on how much their contribution deviated from the group average. The height of the bars indicate the average punishment, and the width is proportional to the number of observations in that category. Our main finding is that only in DEC/1, DEC/0.75, and CEN/1 the size of punishment is overall positively correlated to the absolute size of the deviation from the group average. There is, however, one important difference between treatments DEC and CEN. In the former this overall correlation is driven by a strong partial effect on the deviation, whereas in CEN it is driven by a strong reaction to the absolute size of the contribution.

### 3.4. Reaction to punishment

Table 5 reports the result of three linear regressions of changes in contributions (contribution_*t*_-contribution_*t*−1_) on outcomes in the previous round - one regression each for every level of noise. More specifically, the first estimation (first two columns) includes data from λ = 1 treatments only, and we estimated the average change in contributions for DEC and CEN for six possible outcomes in the previous round. The outcomes in the previous round are identified by (i) whether the participant contributed more than the group average (*above*), the same amount (*equal*), or less (*below*), and (ii) whether she was punished or not. Every regressor is a dummy variable indicating the relevant combination and all calculations are based on actual contributions rather than signals. The maximum likelihood estimations correct for random effects on participants and group^13^. For example, in DEC/1 someone who in the previous period contributed more than the group mean, on average reduces the contribution by 1.025 if there was no punishment and by 1.505 if there was. The table also reports results from one sided Wald tests comparing reactions between institutions (DEC vs. CEN, significance indicated, e.g., by > or ≪) as well as testing for a significant positive effect of punishment on reactions (for example, define δ = “below & punished”-“below & not pun,” then we test H0: δ = 0 vs. H1: δ > 0. Significance is indicated by, e.g., Δ).

**Table 5:** **Change in contributions**.

	λ = 1			λ = 0.75			λ = 0.50		
	**DEC**		**CEN**	**DEC**		**CEN**	**DEC**		**CEN**
**CHANGE IN CONTRIBUTION**									
*D* below & not pun.	1.146^**^	<	2.630^***^	1.220^**^	<	2.734^***^	1.966^***^		2.623^***^
	(2.15)		(5.03)	(2.41)		(6.26)	(4.61)		(6.25)
	Δ		Δ	Δ		Δ	∧		
*D* below & punished	3.681^***^	<	4.968^***^	3.260^***^	≪	4.956^***^	3.067^***^		2.587^***^
	(9.45)		(12.31)	(7.54)		(10.22)	(6.01)		(4.89)
*D* equal & not pun.	−0.394^*^		−0.498^**^	−0.711		−0.54	−0.32		1.063
	(−1.68)		(−2.05)	(−1.29)		(−0.79)	(−0.28)		(1.45)
	⋏					⋏			⋏
*D* equal & punished	0.9	≻	−0.853	−0.947		−2.699^**^	3.053		2.825^***^
	(0.93)		(−1.01)	(−1.02)		(−2.02)	(1.02)		(3.34)
*D* above & not pun.	−1.025^***^		−1.266^***^	−1.900^***^	≫	−3.461^***^	−3.286^***^		−3.937^***^
	(−2.72)		(−4.22)	(−4.77)		(−9.8)	(−8.07)		(−9.62)
			Δ			Δ	∧		
*D* above & punished	−1.505^***^	≪	1.812	−1.734^***^		−1.714^**^	−2.095^***^	>	−3.943^***^
	(−3.19)		(1.61)	(−3.4)		(−2.39)	(−3.46)		(−5.54)
N/Subj./Groups	1856/64/16			1856/64/16			1856/64/16		
chi2	397.1^***^			438.7^***^			448.9^***^		
ll	−5053.7			−5716.8			−5702.7		

Fixed effects margins (t-statistics) from linear maximum likelihood regression with random effect on participant nested in (independent) random effect on matching group.

* p < 0.1,

** p < 0.05,

*** *p < 0.01. Significance of differences between institutions (based on Wald test, one-sided): ≺(p < 0.1), < (p < 0.05), ≪ (p < 0.01). Significance of effect of punishment (based on Wald test, one-sided): ⋏(p < 0.1), ∧ (p < 0.05), Δ (p < 0.01)*.

In both institutions and for all levels of noise, those who in the previous round contributed less, on average significantly increase their contribution, irrespective of whether they were punished or not. In all treatments except CEN/0.5, punishment significantly enhances this reaction. Those who contributed more and were not punished decrease their contributions in all treatment combinations. In DEC, punishment of such cooperative participants has no significant effect on this reaction. In CEN, however, such perverse punishment dampens the reduction, significantly so in CEN/1 and CEN/0.75. Comparing immediate reactions between institutions, treatments CEN/1 and CEN/0.75 in most cases show significantly more cooperative changes in contributions than the equivalent DEC treatments. Note, however, that there are other reactions that span over more periods.

In Table A.5 (Supplementary Material) of the online supplement we report results from similar regressions including the amount of the received punishment for each of the three possible cases (*above* or *below* average or *equal* contribution). Including the amount of the received punishment into the regression increases the shift effect (or “intercept”) throughout, and with only one exception (equal contributors in DEC/1), the reaction to the amount of punishment is negative. For those contributing below average it is significantly negative in all treatments except for CEN/0.75, and for above average contributors it is significantly negative in all except for DEC/0.50 and CEN/0.50.

Result 8. *For negative deviators we find:*

- *Negative deviators increase their contribution in the next period in both institutions*.
- *This effect is stronger if they were punished and punishment was not too high*.
- *A large amount of punishment has a negative effect on contributions*.
- *These immediate reactions are stronger in CEN than in DEC*.

For positive deviators we find:

- *Positive deviators decrease their contribution in the next period*.
- *In DEC punishment has no effect*.
- *In CEN punishment tends to reduce this adjustment*.
- *However, a lot of punishment has a negative effect on contributions in DEC and CEN*.

We furthermore tested whether reactions to punishment differ in early rounds. By and large results are similar and do not differ significantly from the one we reported. The most noteworthy (though insignificant) difference is that in CEN/1 in early rounds, the positive reaction of cooperative types to punishment is much smaller and only later becomes significant.

Finally, to fully assess the question whether punishment has a stronger effect on contribution behavior in DEC or CEN, we compare the change in contribution due to punishment on top of the change in contribution without punishment^14^. More specifically, in both institutions deviators change their contribution even if they are not punished. The effectiveness of punishment is therefore the difference in adjustments. For example, define by Δb (DEC/1) the following differences in coefficients from Table 5. Δb = *(below* & *punished)-(below* & *not pun.)*. We compared these differences between institutions by testing for example *H*0 : Δb (DEC/1) = Δb (CEN/1) against *H*1 : Δb (DEC/1) > Δb (CEN/1). The result of these tests are indicated by appropriate signs between the columns of Table 6.

**Table 6:** **Difference in change in contributions after punishment vs. no punishment**.

	λ = 1			λ = 0.75			λ = 0.50		
	**DEC**		**CEN**	**DEC**		**CEN**	**DEC**		**CEN**
Δbelow	2.535^***^		2.338^***^	2.040^***^		2.222^***^	1.101^**^	≻	−0.036
Δequal	1.294^*^		−0.355	−0.236		−2.159^*^	3.373		1.762^*^
Δabove	−0.480	≪	3.078^***^	0.166	<	1.747^***^	1.191^**^		−0.006

Coefficients and Wald-tests based on results from Table 5. For example: Δ below = (below & punished)-(below & not pun.).

* p < 0.1,

** p < 0.05,

*** *p < 0.01. Significance of differences between institutions: ≺(p < 0.1), < (p < 0.05), ≪ (p < 0.01)*.

Result 9. *For* λ = 1 *and* λ = 0.75*, the effectiveness of punishment on contributions only differs significantly between institutions for positive deviators. For* λ = 0.50*, it differs significantly for negative deviators*.

## 4. Discussion

We test whether centralization of punishment *per se* under *perfect* and *imperfect* information affects behavior and outcomes in voluntary contribution public good games. Both, institution and noisiness of signals are imposed exogenously, and efficiency and cost of punishment are held constant across institutions.

We find significant differences in how contribution behavior is being punished. While centralized authorities do not punish those who contribute more than average, we observe such perverse and furthermore anti-social punishment in the decentralized regime. While in treatments with noise, this perverse punishment is being averaged out, it remains highly relevant if signals are perfect. Otherwise we observe socially reasonable punishment of those who contribute less than the group average in all treatments. Similarly, distributed and received punishment decrease with signal and actual contribution, respectively.

Reactions to punishment by negative deviators are similar between institutions. Even if they are not punished they increase their contribution but punishment enhances this positive adjustment in both institutions by about the same. Positive deviators on the other hand, reduce their contributions in both institutions as long as they were not punished. Interestingly, perverse punishment by their peers has no effect on this reaction in DEC. Thus, while perverse punishment directly induces inefficiencies, it has hardly any consequences in the long run. In CEN on the other hand, where this type of punishment was not as pronounced, it has significant positive effects on contributions.

Despite these differences in behavior, we find that the institution has no effect, neither on contributions, aggregate punishment nor overall efficiency, both if information is perfect (λ = 1) and if there is considerable noise (λ = 0.5). Under intermediate noise (λ = 0.75), however, punishment differs significantly but with only limited consequences for contributions and overall efficiency. More specifically, in the peer to peer punishment institution, punishment escalates during the first third of the experiment keeping contributions higher than under centralized punishment. However, due to the excessive punishment, efficiency is not significantly different.

Anti-social and perverse punishment are frequently observed in public good experiments with peer to peer punishment. In a detailed study of such behavior (Herrmann et al., 2008) suggest dominance, competitive personality, the desire to maximize one's relative payoff, or normative conformity as possible motivating factors. However, there is no obvious reason why these motivating factors should not exist among authorities. The absence of perverse punishment in the CEN treatments therefore suggests that the direct involvement in the contribution stage plays an important role. Alternative explanations such as do-gooder derogation (Monin, 2007) may, however, still apply. Do-gooder derogation requires that one's own uncooperative behavior is illustrated and made worse by the exemplary behavior of the cooperative players. As authorities cannot contribute, they are clearly unaffected by this. Other explanations referring to the repeated interactions, such as counter punishment to prevent future punishment, retaliation, or “feuds” among participants may also explain this asymmetry among institutions. The fact that with increasing noise such punishment behavior becomes less relevant (both in distributed and received punishment) supports this interpretation. The differences in reactions to perverse punishment in DEC and CEN are another piece in the puzzle which is probably due to ascribed motives. Centralized authorities mainly punish cooperators if they wish them to increase their contributions further, whereas under peer-to-peer punishment this is probably not the reason.

What remains difficult to explain is the escalation of punishment in DEC/0.75 which is absent in the equivalent treatment with centralized punishment CEN/0.75. Could it be explained by ruthless satisfaction of punishment sentiments and expression of anger (Dickinson and Masclet, 2015) despite the noise? But why would peers in a decentralized punishment mechanism succumb to such behavior while authorities do not? A possible explanation is that such sentiments are stronger among peers. However, this raises the question why we do not find differences between DEC/1 and CEN/1.

Centralized punishment institutions have been praised in the economic, legal, and political science literature, with recent support from experimental research. However, it is not centralization *per se* that is beneficial but other institutional differences. This issue carries over to studies with *endogenous* institutions, where centralized punishment prevails if it comes with additional advantages (Traulsen et al., 2012), but loses against decentralized punishment in a *ceteris paribus* comparison under perfect information (Grechenig et al., 2015).

## Author contributions

All authors listed, have made substantial, direct and intellectual contribution to the work, and approved it for publication.

## Funding

We gratefully acknowledge financial support from the Max Planck Institute for Research on Collective Goods, Bonn, Germany.

### Conflict of interest statement

The authors declare that the research was conducted in the absence of any commercial or financial relationships that could be construed as a potential conflict of interest. The reviewer DN and handling Editor declared their shared affiliation, and the handling Editor states that the process nevertheless met the standards of a fair and objective review.

## Footnotes

^1^*Anti-social* punishment is directed at someone who contributed more than oneself (see e.g., Herrmann et al., 2008), *perverse* punishment at someone who contributed more than the group average in a symmetric setting (see e.g., Cinyabuguma et al., 2006).

^2^We will use the female genus for the authority and the male genus for the C-participants. The experiments, however, did not control for sex, and no loaded language was used.

^3^Note that due to external validity we decided to keep this informational asymmetry which is a natural feature of these institutions.

^4^The authority's income is reduced by one to guarantee symmetry with treatment *CEN*.

^5^More specifically, we take the average for every matching group and compare the resulting distributions.

^6^Whenever we speak of a “signal,” we are referring to the number the punisher sees on her screen about the contribution of the other participant.

^7^Thus, in CEN the constant of the regression measures average punishment of someone who contributed exactly as much as the group average. Similarly, in DEC it measures the punishment of someone whose contribution does not only match the group average but also equals the contribution of the participant who decides whether to punish.

^8^Furthermore, as Steiner (2007) shows, a punishment rule exclusively targeting the lowest contributor can establish cooperation even if punishment sentiments are very small. It is therefore reasonable to measure cooperativeness in relation to average group contributions.

^9^For this purpose we introduced a dummy variable for institution DEC and interacted it with all variables before running a regression on the combined date for both institutions within one noise treatment.

^10^We thank an anonymous referee for suggesting this analysis.

^11^Although, very difficult to achieve, there could be some form of “tacit” collusion amongst punishers, and participants may learn to identify the accuracy of signals, for example based on repetitive or unique contribution behavior.

^12^It might be surprising that coefficients for CEN/1 in Tables 3, 4 differ. However, in the former, observations are clustered on the authority, compared to the recipient of punishment in the latter.

^13^More specifically, reported reactions are the fixed effects from linear maximum likelihood estimations including independent random effects on participants nested in random effects on matching groups. As in Table 4, variables are defined by actual contributions rather than signals.

^14^We thank an anonymous referee for suggesting this analysis.
