# Supplementary material for: Monopolizing Sanctioning Power under Noise Eliminates Perverse Punishment But Does Not Increase Cooperation
Source: Front Behav Neurosci. 2016 Sep 29;10:180. doi: 10.3389/fnbeh.2016.00180 (PMC5040719; doi:10.3389/fnbeh.2016.00180)
Supplement: Supplementary file 1 [file Presentation1.PDF]

Online Appendix: Monopolizing sanctioning power under noise eliminates perverse punishment but does not increase cooperation

Sven Fischer, Kristoffel Grechenig & Nicolas Meier

August 13, 2016

A. Additional Results

This online appendix presents additional results for the interested reader and the experimental instructions translated from German. The experimental data and programs are also available from the website.

A.1. Descriptives

See Table A.1 for aggregate data relating to Tables 1 and 2.

A.2. Ceiling Effect

In CEN/1 and DEC/1 many contribute fully, such that also average contributions per matching group are close to the ceiling. This clearly creates problems and we can not exclude the possibility that there would be an effect of institution on contributions if no such ceiling existed and those who contribute 20 now, contributed even more. What we can, however, test is whether we would be able to find a significant effect if in one of the institutions even more contributed fully. To test this, we ran several simulations. The outcomes are illustrated in the barplots of Figures A.1a and b; Figure A.1a for data from Periods 1-15 and Figure A.1b for Periods 16-30. Each bar (except for the two to the right at 1.0) represents the outcomes of one thousand simulations. For example, the black bar at 0.6 in Figure A.1a is the outcome of the following procedure: At each of the one thousand simulations we randomly set 60% of contribution decisions in DEC/1 to 20, leaving all other observations unchanged. From this we calculated the averages per matching group over Periods 1-15. We then compared the simulated data for DEC/1 with the actual data in CEN/1 with Wilcoxon signed rank tests and recorded the resulting p-value. As represented by the bar, in about 16.5% of the bootstraps the p-value turned out below 5%. We did the same for nine different probabilities of full contribution ranging from 10% to 90%. Similar results are reported by the grey bars.

Table A.1: Descriptive Statistics

	Medians of Distribution of Matching Group Averages					
	Contributions		Punishment		Efficiency	
Periods	1-15	16-30	1-15	16-30	1-15	16-30
CEN/1	18.5833	19.7167	0.7250	0.0833	11.0933	15.4267
CEN/0.75	12.8333	8.3833	0.8917	0.6750	7.2400	4.5333
CEN/0.50	9.9500	5.7000	0.8000	0.7750	5.0667	2.7400
DEC/1	17.4833	19.7667	0.6333	0.1333	11.4533	15.2800
DEC/0.75	14.7667	15.6333	1.6583	0.8917	1.8867	6.7467
DEC/0.50	9.1333	6.7833	0.7833	0.2333	4.7600	2.5733

	Averages					
	Contributions		Punishment		Efficiency	
Periods	1-15	16-30	1-15	16-30	1-15	16-30
CEN/1	16.0417	17.7083	0.6958	0.1583	10.0500	13.5333
CEN/0.75	13.2000	9.8583	1.0979	0.7917	6.1683	4.7200
CEN/0.50	9.0708	6.1708	0.6958	0.7563	4.4733	1.9117
DEC/1	15.9917	16.3583	0.8750	0.2500	9.2933	12.0867
DEC/0.75	13.8375	14.5542	2.0521	1.0146	2.8617	7.5850
DEC/0.50	8.9708	6.5500	0.9250	0.4563	3.4767	3.4150

Notes: Reported medians are based on the distribution of the averages per matching group over the relevant time span.

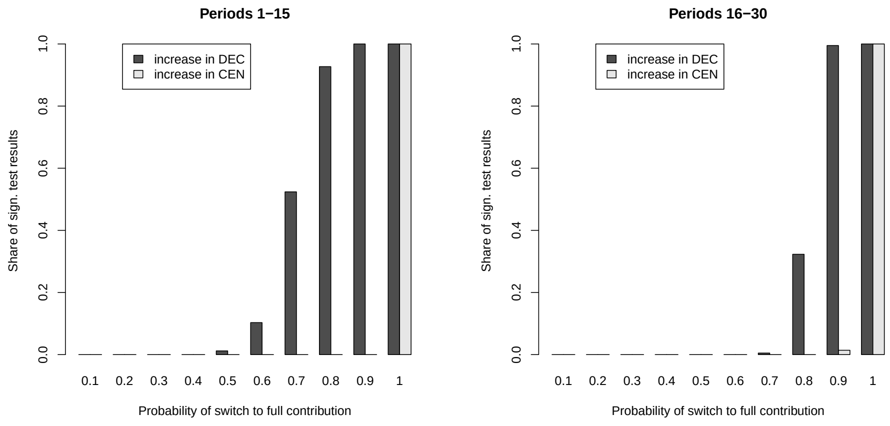

(a) First Half $\alpha = 5\%$ (b) Second Half $\alpha = 5\%$

Figure A.1: Simulation: Significant differences in contributions between DEC/1 and CEN/1

Note: Bars represent share of significant differences in contributions between DEC/1 and CEN/1 following simulation of various likelihoods of contributing fully in one of the institutions. Numbers on x-axis are the share of contributions in either DEC (black bars) or CEN (grey bars) that are simulated to be 20.

Here, however, we simulated more full contribution in CEN/1 rather than DEC/1. The bars to the right at 1.0 are based on one simulation each, where we simply set all contributions in CEN/1 (black bar) or DEC/1 (grey) to 20.

Results clearly indicate that it would have been possible to find a significant effect of institution on contributions, if only enough contributed fully in DEC or alternatively all contributed fully in CEN. We did the same analysis with an acceptance threshold of 5%. This clearly resulted in less significant findings. Still, in both parts of the experiment it is reasonably possible to find a significant effect.

A.3. Distributed Punishment

See Table A.2 for an extended model explaining how punishment is distributed.

A.4. Received Punishment

Tables A.3 and A.4 report the results of mixed effects poisson regressions of the received punishment on several regressors. The estimations extend the one reported in Table 4 by including the continuous variables *below* and *above*, which measure the exact deviation from the average group contribution in absolute terms. More specifically, if c_i denotes participant i's contribution and $\overline{c}$ the average group contribution, then $below_i = \overline{c} - c_i$ if $c_i < \overline{c}$ and $below_i = 0$ otherwise. Similarly, $above_i = c_i - \overline{c}$ if $c_i > \overline{c}$ and $above_i = 0$ otherwise. While in Table A.4 we control for effects of the absolute size of Contribution

Table A.2: Distributed Punishment

Treatment	(1) DEC/1	(2) DEC/.75	(3) DEC/.50	(4) CEN/1	(5) CEN/.75	(6) CEN/.50
distributed punishment points						
Signal	-0.0124	-0.0115	-0.211***	-0.214***	-0.140***	-0.0234
	(-0.32)	(-0.33)	(-6.07)	(-6.13)	(-3.17)	(-0.28)
*D*Freerider	-0.115	-0.0172	-0.211	-0.0247	0.272	0.243
	(-0.42)	(-0.07)	(-1.36)	(-0.06)	(0.66)	(0.67)
*D*belowGroup	2.034**	1.229***	0.517	3.212***	1.067	0.715
	(2.12)	(4.77)	(1.52)	(5.52)	(1.54)	(0.76)
belowGroup	0.146***	0.0610	-0.0263	-0.131***	-0.0636	0.152***
	(5.15)	(1.45)	(-0.73)	(-3.28)	(-0.99)	(4.37)
*D*aboveGroup	0.761*	0.606**	0.100	1.510**	-0.167	1.232
	(1.81)	(2.08)	(0.52)	(1.97)	(-0.26)	(1.33)
aboveGroup	0.255***	-0.229***	0.0684	-0.714***	0.0759	-0.00646
	(3.16)	(-6.71)	(1.37)	(-3.66)	(0.45)	(-0.08)
*D*less	1.107	1.535***	0.414*			
	(1.57)	(8.34)	(1.88)			
less	0.0405	0.0302***	0.0166			
	(1.17)	(3.20)	(0.85)			
*D*more	1.067*	1.011***	-0.0211			
	(1.92)	(3.48)	(-0.16)			
more	0.0276	0.0676	0.134***			
	(1.32)	(1.16)	(4.83)			
Period	0.217	0.160	-0.254	0.851***	0.427**	-0.974**
	(1.03)	(0.77)	(-1.06)	(3.94)	(1.99)	(-1.98)
_cons	-5.634***	-3.817***	-1.807**	-1.267	-0.610	-0.163
	(-5.02)	(-5.87)	(-2.12)	(-1.45)	(-0.41)	(-0.09)
N(#Groups)	2880(8)	2880(8)	2880(8)	960(8)	960(8)	960(8)
ll	-848.6	-1906.1	-1111.0	-384.1	-1127.1	-1037.8
Wald chi2	884.9***	1390.5***	530.2***	920.3***	956.2***	499.7***

Poisson regression of distributed punishment points with random shift effect on matching group and subject (DEC only, random effects independent). All regressors are defined positively such that a positive (negative) coefficient implies increasing (decreasing) punishment. Not reported: fixed effect on every Period (following Period 2). z statistics in parentheses based on Huber White standard errors (not Wald chi2). Significance: $^{*}(p < 0.1)$, $^{**}(p < 0.05)$, $^{***}(p < 0.01)$.

and indicator variable Freerider, in Table A.3 we do not. Figure 2 in the main document illustrates how received punishment is correlated to the deviation of the contribution to the group average.

Table A.3: Received Punishment extended 1

Treatment	(1) DEC/1	(2) DEC/.75	(3) DEC/.50	(4) CEN/1	(5) CEN/.75	(6) CEN/.50
deducted income						
*D*belowGroup	2.701***	0.552*	0.0276	3.238***	0.5947**	0.2817
	(421)	(1.75)	(0.03)	(3.94)	(3.03)	(1.01)
below	0.2117***	0.0810***	0.0506*	0.1405***	0.0674**	0.0257
	(6.45)	(3.58)	(1,71)	(7.23)	(2.26)	(0.64)
*D*aboveGroup	1.6022***	0.0241	0.2223	1.573*	-.3087	-0.3287
	(4.23)	(0.11)	(0.33)	(1.65)	(-0.95)	(-0.94)
above	0.2057**	-0.0554	-0.0874*	-0.797***	0.024	0.0298
	(2.21)	(-1.37)	(-2.58)	(-5.56)	(0.28)	(0.49)
Period	0.0427***	0.0018	-0.0508	-0.0340	0.0023	-0.0958
	(2.91)	(0.09)	(-1.01)	(-1.54)	(0.04)	(-1.52)
_cons	-2.812***	1.259***	0.3141	-1.8870**	-0.2712	0.5277
	(-3.58)	(4.16)	(0.29)	(-2.12)	(-0.28)	(0.47)
N(#Groups)	960(8)	960(8)	960(8)	960(8)	960(8)	960(8)
Wald chi2	1871.58***	1766.29***	540.78***	2013.10***	1258.93***	753.26***
ll	-1113.1	-4718.8	-2124.1	-864.6	-3652.2	-2779.0

Mixed effect poisson regression of received punishment with random shift effect on subject and matching group (independent of each other). All regressors are positively defined, such that a positive (negative) coefficient implies increasing (decreasing) punishment. Not reported: fixed effect on every Period (following Period 2). Huber White standard errors (not for Wald chi2). z statistics in parentheses. Significance: $^{*}(p < 0.1)$, $^{**}(p < 0.05)$, $^{***}(p < 0.01)$.

Let us first look at contributions below the group average. In both DEC/1 and DEC/.75 the punishment a negative deviator receives significantly increases with the absolute deviation. In CEN/1 we find a similar overall correlation, i.e. someone who contributed less than average receives more punishment the more he deviated (TableA.3). However, once we include the actual level of contribution into the regression (Table A.4), the coefficient on *belowGroup* changes its sign and the effect on the level of contribution turns out very large. The two opposing effects are together significantly positive. The overall correlation is therefore primarily driven by the absolute level of the contribution, not the distance to the group average. In all other treatments (DEC/.50, CEN/.75, and CEN/.50) there is no significant reaction to the size of the deviation.

RESULT A1:

- *In DEC/1 and DEC/.75, everything else equal, negative deviators are punished*

Table A.4: Received Punishment extended 2

Treatment	(1)<br>DEC/1	(2)<br>DEC/.75	(3)<br>DEC/.50	(4)<br>CEN/1	(5)<br>CEN/.75	(6)<br>CEN/.50
deducted income						
Contribution	0.00881	0.0354**	-0.0560*	-0.229***	-0.00313	-0.0628
	(0.29)	(2.04)	(-1.81)	(-6.34)	(-0.13)	(-1.31)
DFreerider	-0.0127	-0.144	0.333*	0.132	1.062***	0.281
	(-0.04)	(-0.63)	(1.77)	(0.25)	(5.60)	(0.88)
DbelowGroup	2.733***	0.604*	-0.145	2.995***	0.533**	0.478***
	(3.76)	(1.90)	(-0.16)	(5.07)	(2.17)	(2.79)
below	0.219***	0.133***	-0.0106	-0.128***	-0.00214	-0.0300
	(6.73)	(4.96)	(-0.21)	(-4.51)	(-0.07)	(-0.54)
DaboveGroup	1.634***	0.0481	0.165	1.705**	-0.341	0.0538
	(4.64)	(0.21)	(0.25)	(2.30)	(-1.10)	(0.22)
above	0.192***	-0.0707*	-0.0241	-0.760***	0.0530	0.0973
	(3.32)	(-1.80)	(-0.40)	(-4.02)	(0.76)	(1.35)
Period	0.0447**	-0.00681	-0.0661	0.0380**	-0.0159	-0.125*
	(2.11)	(-0.33)	(-1.47)	(2.38)	(-0.37)	(-1.68)
_cons	-2.965***	0.765*	0.966	1.015	0.136	0.880
	(-2.73)	(1.91)	(0.83)	(1.08)	(0.24)	(0.66)
N(#Groups)	960(8)	960(8)	960(8)	960(8)	960(8)	960(8)
Wald chi2	1868.5***	1806.1***	610.9***	2081.2***	1634.2***	822.3***
ll	-1112.6	-4699.8	-2088.5	-775.8	-3515.8	-2744.3

Mixed effect poisson regression of deducted income with random shift effect on subject and matching group (independent of each other). All regressors are positively defined, such that a positive (negative) coefficient implies increasing (decreasing) punishment. Not reported: fixed effect on every Period (following Period 2). Huber White standard errors (not for Wald chi2). z statistics in parentheses. Significance: $^{*}(p < 0.1)$, $^{**}(p < 0.05)$, $^{***}(p < 0.01)$.

more, the more they deviate from the group.

- *In CEN/1 negative deviators overall receive more punishment the further away they are from the group average. However, contrary to DEC/1, this effect is driven by strong punishment of small contributions, rather than by a reaction to the deviation from the group. The latter partial effect even opposes the overall correlation.*

In Table 4 of the main document we found evidence for perverse punishment in DEC/1. This is also reflected in a significant positive partial effect, i.e. perverse punishment increases with increasing positive deviation from the group average. In DEC/.75 and CEN/1 this partial effect is significantly negative, and in DEC/.50, CEN/.75 and CEN/.50 it is insignificant. We checked the robustness of all these effects by stepwise elimination of insignificant regressors as well as by introducing other variables such as, e.g. the average sum of contributions in the group over all previous periods. All the reported results were confirmed.

Result A2:

- *In DEC/1, everything else equal, punishment of positive deviators increases with the size of the deviation from the group average.*

A.5. Reaction to Punishment

Table A.5 reports results from three linear regression of changes in contributions (contribution$_t$-contribution$_{t-1}$) on outcomes in the previous round, one each for every level of noise. More specifically, the first estimation (reported in the first two columns) includes data from DEC/1 and CEN/1 and we estimated the average change in contributions for DEC and CEN separately for several possible outcomes in the previous round. The outcomes in the previous round are identified by dummy variables which indicate i) whether the participant contributed more than the group average (*above*), the same amount (*equal*), or less (*below*), and ii) whether she was punished or not. Furthermore, if a participant was punished, we regress on the amount of the received punishment. For this we include variables *above: punishment* and *above: punishment* which consist of the amount of punishment in the relevant situation and are zero otherwise. The maximum likelihood estimations correct for random effects on participants and group.

B. Translated Instructions

The following is a translation of the German instructions. The main text is taken from treatment *DEC*/1. Where instructions differed between treatments we highlight this.

General Instructions

Table A.5: Change in Contributions

	$\lambda = 1$ DEC		$\lambda = 1$ CEN	$\lambda = 0.75$ DEC		$\lambda = 0.75$ CEN	$\lambda = 0.50$ DEC		$\lambda = 0.50$ CEN
change in contribution									
D below & not pun.	1.243**	$<$	2.960***	1.229**	$<$	2.691***	1.963***		2.620***
	(2.33)		(5.62)	(2.47)		(6.28)	(4.66)		(6.31)
D below & punished	5.469***	$<$	6.776***	4.020***	$\prec$	5.198***	3.624***		3.142***
	(12.35)		(14.77)	(8.22)		(9.92)	(6.2)		(5.41)
below: punishment	-0.351***	$>$	-0.520***	-0.108***		-0.0703	-0.131**		-0.191**
	(-8.64)		(-8.1)	(-3.16)		(-1.27)	(-1.96)		(-2.33)
D equal & not pun.	-0.409		-0.614**	-0.741		-0.543	-0.297		1.028
	(-1.58)		(-2.3)	(-1.37)		(-0.81)	(-0.26)		(-1.41)
D equal & punished	-0.0372		0.135	-0.645		-0.947	5.351		3.018***
	(-0.02)		(0.15)	(-0.64)		(-0.59)	(1.46)		(3.31)
D equal: rec. punishment	0.294		-0.152**	-0.108		-0.453*	-2.31		-0.135
	(0.71)		(-2.06)	(-0.71)		(-1.91)	(-1.1)		(-0.72)
D above & not pun.	-1.061***		-1.215***	-1.883***	$\gg$	-3.422***	-3.256***		-3.900***
	(-2.76)		(-3.83)	(-4.83)		(-9.91)	(-8.09)		(-9.65)
D above & punished	-0.303	$\ll$	3.450**	-0.981		-0.274	-1.725**	$>$	-3.677***
	(-0.5)		(2.46)	(-1.63)		(-0.37)	(-2.44)		(-4.75)
above: punishment	-0.264***		-0.396*	-0.132**	$\gg$	-0.468***	-0.0958		-0.102
	(-3.43)		(-1.72)	(-2.21)		(-5.84)	(-1.01)		-0.8
N/Subj./Groups	1856/64/16			1856/64/16			1856/64/16		
chi2	608.2***			504.4***			462.2***		
ll	-4978.4			-5689.9			-5696.4		

Fixed effects margins (z-statistics) from linear maximum likelihood regression with random effect on participant nested in (independent) random effect on matching group. Significance: *($p < 0.1$), **($p < 0.05$), ***($p < 0.01$). Significance of differences between institutions (based on Wald test, one-sided): $\prec$ ($p < 0.1$), $<$ ($p < 0.05$), $\ll$ ($p < 0.01$).

You are about to take part in an economic experiment. If you read the following instructions carefully, you can earn a substantial amount of money, depending on the decisions you make. It is therefore very important that you read these instructions carefully.

The instructions you have received from us serve your own private information only. During the experiment, any communication whatsoever is forbidden. If you have any questions, please ask us. Disobeying this rule will lead to exclusion from the experiment and from any payments. During the experiment, we speak not of Euro, but instead of Taler. Your entire income is hence initially calculated in Taler. The total number of Taler you earn during the experiment is converted into Euro at the end, at the rate of

$$1 \text{ Taler} = 1 \text{ Eurocent.}$$

At the end, you will be paid in cash the amount of Taler you have earned during the experiment, in addition to 2.50 Euro for taking part.

The experiment is divided into different periods. In total, there are 30 periods. Participants are divided into groups of five, so your group has another four participants, plus yourself. During these 30 periods, the constellation of your group of five remains unchanged. You are therefore in the same group with the same participants for 30 periods. In each period, you and the other participants in your group will be assigned a random identification number. Please note, however, that this number changes randomly in each round. Group members are therefore not identifiable beyond the respective periods. At the beginning, each of the five participants is randomly assigned a role for the duration of the entire experiment. Four participants make decisions in the role of A, and one in the role of B. You keep your role during the entire experiment.

The exact procedure of the experiment is described on the following pages.

Exact Procedure of the Experiment

Each of the 30 periods has two stages.

Stage 1: Contribution to the Project

Participant A:

At the beginning of each period, each of the four A participants receives an endowment of 20 Taler. Each participant A has to decide how many of the 20 Taler to keep and how many to contribute to the project. All even numbers are possible contributions, i.e., 0, 2, 4, 6, . . ., 18, 20. All A participants in your group make their respective decisions simultaneously and independently.

After this, the incomes from Stage 1 are calculated:

For each Taler that you keep, you receive exactly one Taler. For each Taler that you and the other participants have invested in the project, each participant receives 0.4 Taler. (Every Taler you invest in the project hence raises the income of each A

participant by 0.4 Taler. Conversely, every Taler another participant has invested in the project raises your own income by 0.4 Taler):

Your income from Stage 1 (Participant A) is:<br>+20<br>- your contribution to the joint project<br>+ 0.4× total sum of contributions to the project

The income from the project is calculated by this formula for all four group members.

Participant B:

Participants in role B do not receive any endowment, nor can they contribute anything to the project. A participant B receives 0.4 times the total sum of the contributions to the project. (For every Taler a participant A invests in the project, participant B hence pays 0.4 Taler):

Your income from Stage 1 (Participant B) is:<br>+ 0.4× total sum of the contributions to the project

Please note: Each of the five participants of a group draws the same income from the project, namely 0.4 times the total sum of the contributions to the project, independently of the role they played and of what they invested.

Stage 2: Points Subtracted

Information:

At the beginning of Stage 2, all participants (roles A & B) are informed about the contributions of the (other) A participants to the project.

[`Additional Instructions for DEC/0.5 and CEN/0.5:`
This information (called a "signal") has a 50% chance of being correct. In other words, in 5 out of 10 cases, the number corresponds to the exact contribution. In the other 5 out of 10 cases, participants see just another random number that does not correspond to the exact contribution. Any other number apart from the exact contribution has the same chance of appearing; further, all participants receive the same information - except for the person whose contribution is being dealt with - and hence see the same number.]

In stage 2 of every round, all *A* participants receive an additional 10 Taler. The *B* participant receives 40 Taler in the second stage of every round.

Distribution of subtraction points:
Each participant *A* can reduce the income of other *A* participants by distributing up to 10 subtraction points. Each of these subtraction points, given by one participant *A* to another, reduces the latter's income by 3 Taler. Similarly, each subtraction point distributed costs the distributor 1 Taler and the participant *B* a further Taler. You keep all subtraction points that have not been distributed.

Income from the Round:

Round income (stages 1 & 2) of participant *A*:
+ income from stage 1
+ 10 Taler (additional endowment)
- 3 × sum of subtraction points received from other *A* participants
- sum of subtraction points distributed to other *A* participants

Round income (stages 1 & 2) of a participant *B*:
+ income from stage 1
+ 40 Taler (additional endowment)
- sum of subtraction points distributed by participants *A* to other *A* participants

[`Different Instructions for CEN/1 and CEN/0.5:`

Distribution of subtraction points:
By distributing up to 40 subtraction points, participant *B* can reduce the income of participants of role *A*. Every subtraction point distributed to an *A* participant by participant *B* reduces *A*'s income by 3 Taler. At the same time, each subtraction point distributed costs participant *B* 1 Taler, and every other participant *A* 1/3 Taler (except the one who received the subtraction point). *B* can give a single *A* participant a maximum of 30 penalty points. You keep all subtraction points that have not been distributed.

Income from the Round:

Round income (stages 1 & 2) of participant A:
+ income from stage 1
+ 10 Taler (additional endowment)
- $3\times$ sum of subtraction points received from participant B
- $1/3\times$ sum of subtraction points distributed by participant B to other A participants

Round income (stages 1 & 2) of a participant B:
+ income from stage 1
+ 40 Taler (additional endowment)
- sum of subtraction points distributed by participant B to other A participants

]

`Same Instructions all treatments:` **Information at the End of the Round and Total Income:**

At the end of each round, you receive a detailed overview of your income from the round: Taler you kept, your income from the project and the resulting income from stage 1, the cost of the subtraction points, the resulting income reduction, the period income.

Your total income at the end of the experiment is the sum of the period incomes.

Anything unclear? Please ask one of the supervisors!

Figure B.1: Screenshots Punishment, DEC & CEN

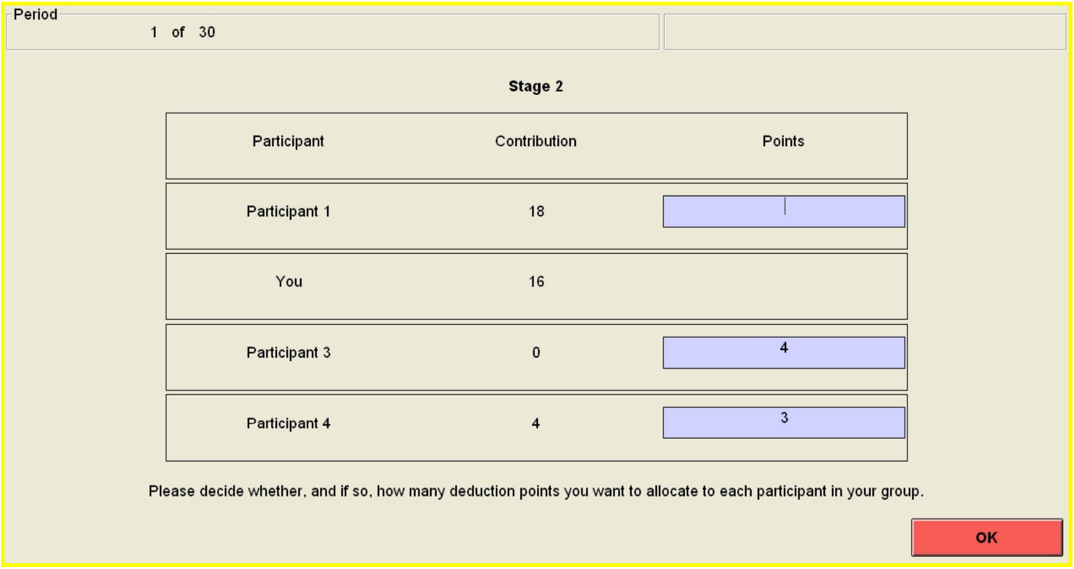

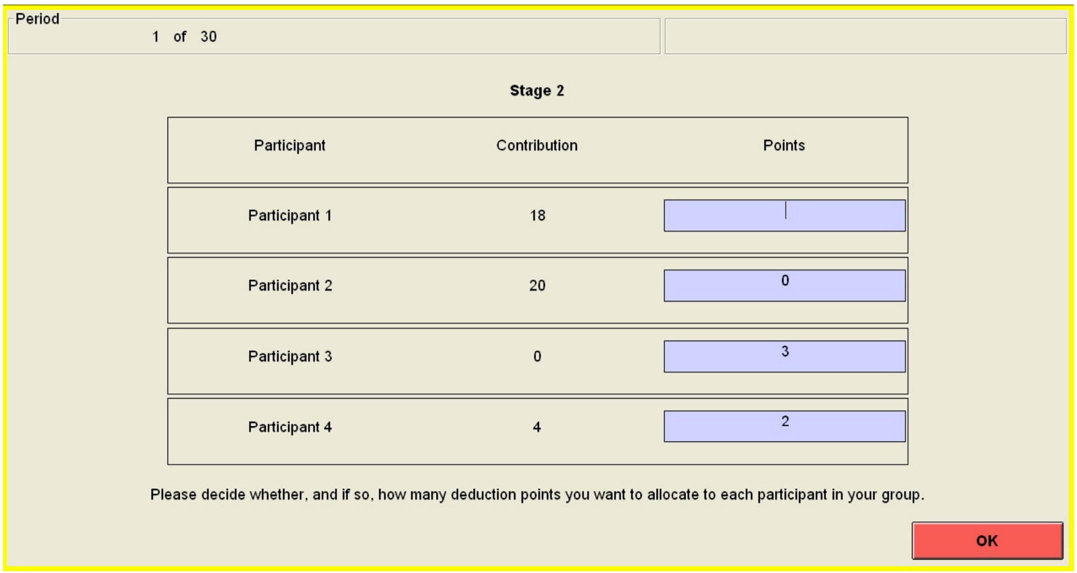

Note: English translation - original program was in German.
