# Supplementary figures and images for: Monopolizing Sanctioning Power under Noise Eliminates Perverse Punishment But Does Not Increase Cooperation
Source: Front Behav Neurosci. 2016 Sep 29;10:180. doi: 10.3389/fnbeh.2016.00180 (PMC5040719; doi:10.3389/fnbeh.2016.00180)

## Contributions

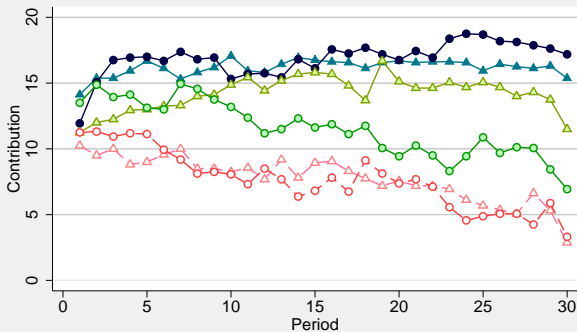

## Punishment

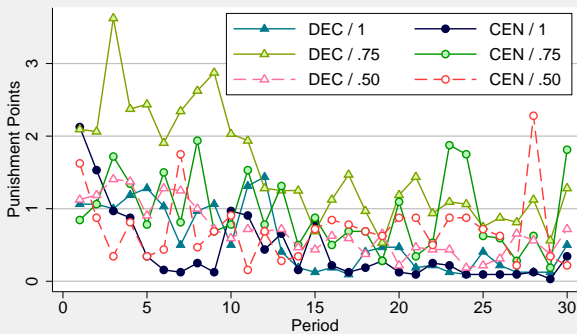

## Efficiency

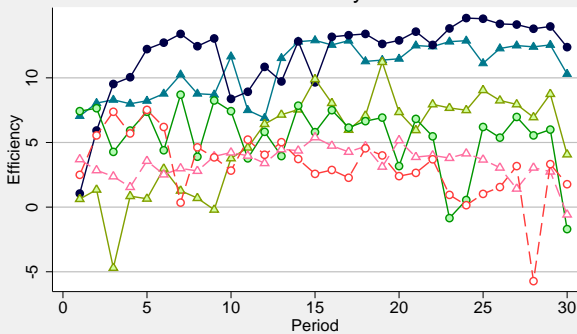

Supplement: Supplementary file 4 [file Image1.PDF]
