# Supplementary material for: Monopolizing Sanctioning Power under Noise Eliminates Perverse Punishment But Does Not Increase Cooperation
Source: Front Behav Neurosci. 2016 Sep 29;10:180. doi: 10.3389/fnbeh.2016.00180 (PMC5040719; doi:10.3389/fnbeh.2016.00180)
Supplement: Supplementary file 5 [file Image2.PDF]

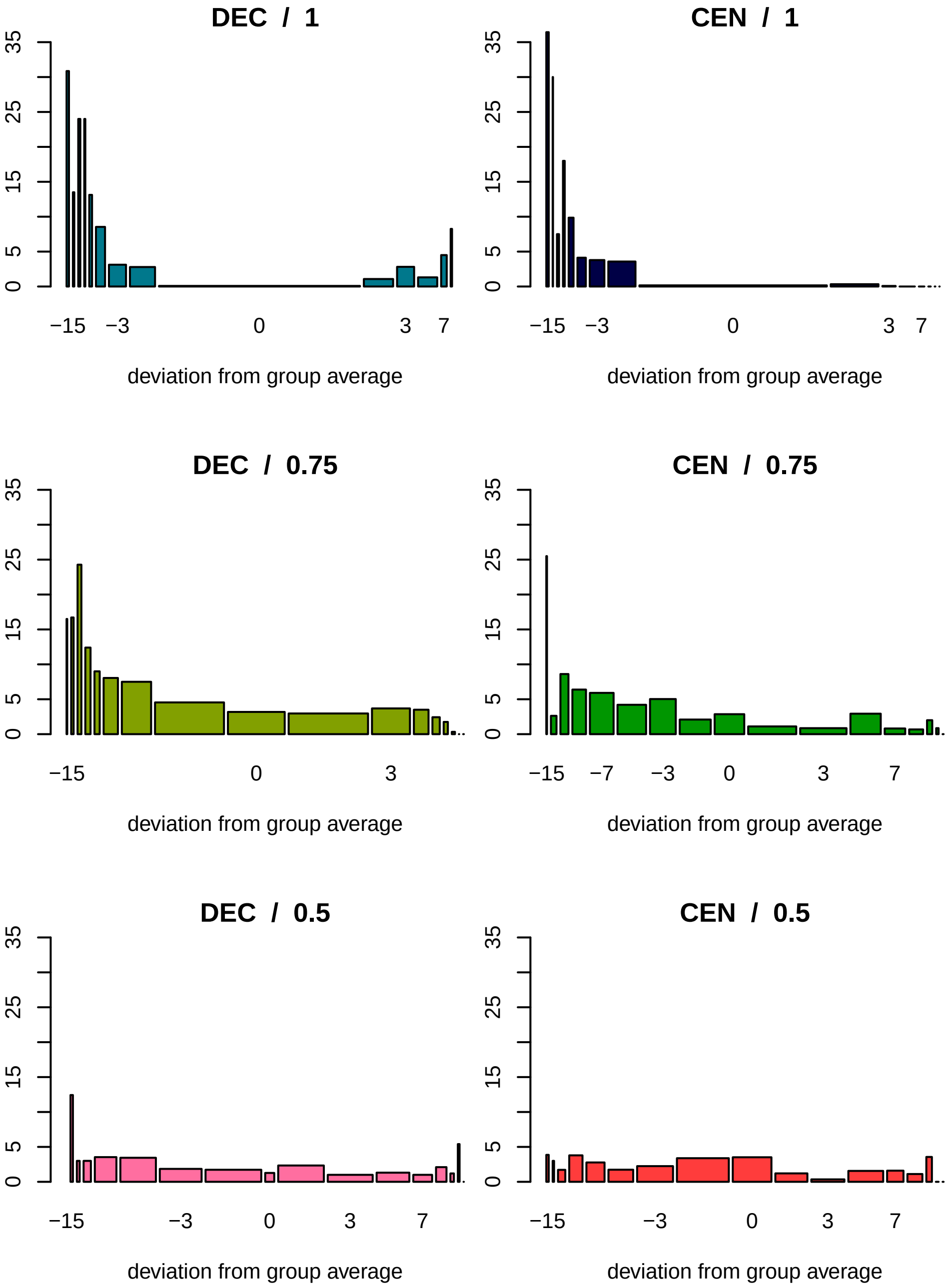
DEC / 1
CEN / 1
DEC / 0.75
CEN / 0.75
DEC / 0.5
CEN / 0.5
0
5
15
25
35
−15
−7
−3
0
3
7
deviation from group average
